# Establishment of a Homologous Silencing System with Intact-Plant Infiltration and Minimized Operation for Studying Gene Function in Herbaceous Peonies

**DOI:** 10.3390/ijms25084412

**Published:** 2024-04-17

**Authors:** Kaijing Zhang, Xiaobin Wang, Xiaoxuan Chen, Runlong Zhang, Junhong Guo, Qiyao Wang, Danqing Li, Lingmei Shao, Xiaohua Shi, Jingtong Han, Zhiyang Liu, Yiping Xia, Jiaping Zhang

**Affiliations:** 1 Genomics and Genetic Engineering Laboratory of Ornamental Plants, Department of Horticulture, Institute of Landscape Architecture, College of Agriculture and Biotechnology, Zhejiang University, Hangzhou 310058, China; jing1998@zju.edu.cn (K.Z.); xiaobinwang@zju.edu.cn (X.W.); 22316296@zju.edu.cn (X.C.); zhangrunlong@sjtu.edu.cn (R.Z.); 22216291@zju.edu.cn (J.G.); 22216279@zju.edu.cn (Q.W.); danqingli@zju.edu.cn (D.L.); 12116125@zju.edu.cn (L.S.); 3200100100@zju.edu.cn (J.H.); ypxia@zju.edu.cn (Y.X.); 2 Zhejiang Institute of Landscape Plants and Flowers, Hangzhou 311251, China; xiaohuashi0826@gmail.com; 3 Harbin Academy of Agricultural Sciences, Harbin 150029, China; liuzhiyanger@126.com

**Keywords:** gene function, silencing, VIGS, herbaceous peonies, *Paeonia lactiflora*, one-year-old roots, intact-plant infiltration, minimized operation

## Abstract

Gene function verification is a crucial step in studying the molecular mechanisms regulating various plant life activities. However, a stable and efficient homologous genetic transgenic system for herbaceous peonies has not been established. In this study, using virus-induced gene silencing technology (VIGS), a highly efficient homologous transient verification system with distinctive advantages was proposed, which not only achieves true “intact-plant” infiltration but also minimizes the operation. One-year-old roots of the representative species, *Paeonia lactiflora* Pall., were used as the materials; prechilling (4 °C) treatment for 3–5 weeks was applied as a critical precondition for *P. lactiflora* to acquire a certain chilling accumulation. A dormancy-related gene named *HOMEOBOX PROTEIN 31* (*PlHB31*), believed to negatively regulate bud endodormancy release (BER), was chosen as the target gene in this study. GFP fluorescence was detected in directly infiltrated and newly developed roots and buds; the transgenic plantlets exhibited remarkably earlier budbreak, and *PlHB31* was significantly downregulated in silenced plantlets. This study established a homologous transient silencing system featuring intact-plant infiltration and minimized manipulation for gene function research, and also offers technical support and serves as a theoretical basis for gene function discovery in numerous other geophytes.

## 1. Introduction

Research on gene function is crucial for revealing the molecular mechanisms regulating plant growth, development, and environmental adaptation. The technological means for verifying gene function include overexpression, knockout, and silencing [1]. Establishing a stable and efficient homologous verification system for the target plant is a fundamental precondition for in-depth research on genes related to various life activities of plants [2]. However, constructing such a system rapidly for every plant species presents a significant challenge for scientists because numerous species exhibit significant species differences. Consequently, scientists can only make speculations regarding gene function based on multi-omics results or gene expression patterns, and then have to adopt model plants such as Arabidopsis and poplar for heterogenic verification. These studies are not sufficiently convincing compared to homologous verification with the target plants themselves, and have thus restricted further exploration of the mechanism of the target plants, such as herbaceous peonies [3].

**Figure 1:**
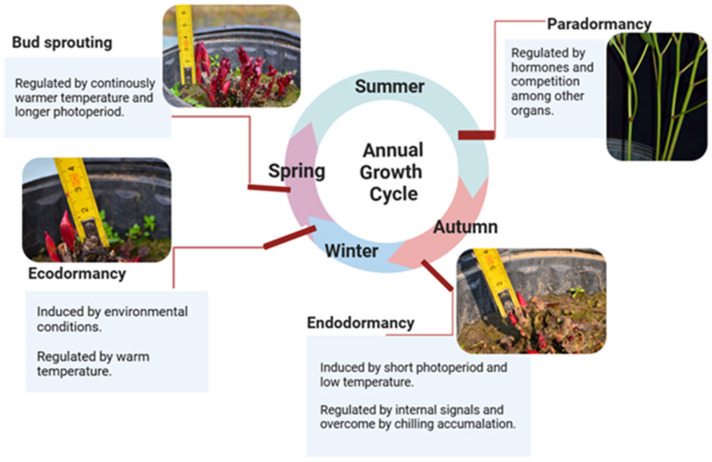
Annual growth cycle of *P. lactiflora* Pall., a representative species of herbaceous peonies in the family Paeoniaceae.

Herbaceous peonies belong to the genus *Paeonia* of the family Paeoniaceae and include more than 30 herbaceous species worldwide, most of which are world-renowned plants with excellent ornamental, medicinal, and edible values [4]. Herbaceous peonies are all geophytes with underground buds generated on the rhizomes or tuber roots (such as *P. anomala* L). With the coming of autumn, the aboveground parts of herbaceous peonies entirely wither and disappear, whereas the underground buds enter the endodormancy stage and can be released after several months of chilling accumulation in winter, namely the fulfilment of chilling requirements (CRs) [5,6,7,8]. After this crucial stage, known as bud endodormancy release (BER), buds transition into the ecodormancy stage, concluding as temperatures increase and day length extends. Finally, buds sprout and tender stems and leaves proliferate in spring; then, plants more than three years old can bloom approximately 40–70 days after bud sprouting [9] (Figure 1).

Undoubtedly, *P. lactiflora* Pall. is the most famous species of herbaceous peony due to its beautiful flowers, elegant leaves, and remarkable pharmaceutical roots. Therefore, this species is specially addressed as the singular “herbaceous peony” and is most widely cultivated in Europe, North America, Australia, and Asia [10]. *P. lactiflora* has often been selected as a representative species in scientific research on herbaceous peonies [11]. However, a complete, stable, and efficient homologous transgenic system has not yet been established for *P. lactiflora* and all other herbaceous peonies because the traditional tissue culture of herbaceous peonies faces numerous hurdles, such as severe callus browning and vitrification, contamination, poor induction and proliferation efficiencies, and low survival rates after transplantation and during acclimation [12,13,14,15]. All these problems seriously hinder scientists from validating the function of the genes that regulate various physiological activities of herbaceous peonies.

The virus-induced gene silencing (VIGS) technique has become increasingly reliable for identifying gene functions in various plants over the last decade. This reverse genetic technique adopts viral vectors carrying a target gene fragment to produce dsRNA and then triggers RNA-mediated gene silencing [16]. One of the most widely used viral delivery vectors is tobacco rattle virus (TRV) due to its simple expression in meristematic tissue [17]. VIGS has many advantages compared with other loss-of-function approaches because it allows phenotypes to achieve rapid performance, provides time-saving experimental processes, does not require complex tissue culture, and has the potential to silence either individual or multiple members of a gene family [18]. VIGS has been used for many plant species to characterize the genes involved in various plant development and stress tolerance processes [19,20].

Previous studies have focused on gene function research using VIGS, but these efforts have yielded incomplete results in recent years. Tree peonies are a group of woody species in the family Paeoniaceae, and they and herbaceous peonies are generally collectively referred to as “peonies”. The silencing of flavonoid glycosyltransferase genes (*UFGTs*) and rooting-related oxygenase (*ARRO-1*) by VIGS in a tree peony, i.e., *P. suffruticosa* (a species of tree peonies), showed that the two genes play key roles in flower color formation and root development [21,22]. The *TRYPTOPHAN DECARBOXYLASE* gene (*TDC*) and *WRKY41*a are highly expressed in stems of *P. lactiflora*, and their silencing by VIGS decreases the secondary wall thickness and distinctly weakens the stem strength. *PlWRKY65*-silenced plants of *P. lactiflora* are more sensitive to *Alternaria tenuissima* infection and show significantly reduced disease resistance compared to wild-type plants [23,24,25]. Research on thermotolerance has shown that the heat stress tolerance of *P. lactiflora* transgenic plants with the transient silencing of mitogen-activated protein kinase 1 (*MAPK1*) by VIGS is significantly enhanced in contrast to that of the control plants [26]. Three-year-old roots of herbaceous peonies have been used as materials to identify the function of *PlDELLA* during the endodormancy stage via VIGS, and the results suggested that *PlDELLA* might negatively regulate BER in *P. lactiflora* [27]. This study represents the first application of gene silencing techniques to investigate bud dormancy in peonies. Subsequently, the dual functions of microRNA172 (miR172) and the *TARGET OF EAT* (*TOE3*) module and PsRGL1 (RGA-LIKE1) in regulating BER were detected by silencing these target genes in the inner flower buds of tree peony, and the results showed that these two genes probably play opposite regulatory roles in the dormancy release of flower buds [28,29].

From the abovementioned examples, it can be seen that only a few studies have reported the gene functions identified by VIGS and the infected organs are totally different, indicating that this technique remains immature in research on peonies. To explore gene functions in peonies using VIGS, an intact plant remains the optimal material for infection, phenotype observation, and gene function validation, although in vitro organs such as petals and flower buds could also be used as infection materials for gene silencing and to verify their functions to a certain degree [21,28]. For example, the dormancy of in vitro inner flower buds without outer scales in MS medium cultivation is not exactly equivalent to the dormancy of mixed buds generated on underground rhizomes of an intact plant in field or pot cultivation. Therefore, the functional identification of the same gene using in vitro organs cannot be regarded as completely equivalent to that using a natural intact plant.

However, “intact-plant infiltration/silencing” is difficult to implement in herbaceous peonies. Taking bud dormancy research as an example, three-year-old roots of *P. lactiflora* are bulky and thick; thus, many problems related to cleaning and disinfection and their high contamination rate are all unavoidable. In addition, at least 4 L of infection solution and sterile water and a sufficiently large container are required for each root during the infection process [27]. In most cases, many roots are needed as replicates. Thus, an adequately large operation space, a sufficient mass of experimental material and enough manpower are required to complete such an inconvenient operation. Therefore, the establishment of a VIGS system with minimized operation and intact-plant infiltration is of great value in studying gene functions in herbaceous peonies.

In this study, a homologous transient silencing system based on VIGS was established, and this system not only realizes intact-plant infiltration but also requires minimized manipulation, materials, and manpower. This study not only establishes an innovative and efficient operational approach for gene function identification in more than 30 herbaceous peony species but also provides a valuable reference for the homology verification of gene function in all geophytes without a stable homologous genetic transformation system.

## 2. Results

### 2.1. Construction of the TRV2-GFP-PlHB31 Recombinant Vector

The inserted fragment of *PlHB31* was cloned by PCR amplification; the products were detected through electrophoresis, and the band length was approximately 274 bp. Monocolonies of *E. coli DH5α* were then obtained for DNA sequencing. The nucleotide sequence of the recombinant vector, including the inserted fragment of *PlHB31*, was confirmed by sequence alignment, and the *PlHB31* fragment was successfully inserted between the two restriction sites, with only one mismatch between nucleotide bases (Figure S1). The positive plasmid was then transformed into GV3101 for subsequent infection, and monocolonies were clearly observed on Luria–Berta medium plates containing 50 mg/L kanamycin (LBK). The PCR products of the recombinant plasmid could be amplified from Agrobacterium monocolonies, indicating the success of the vector construction and transformation.

### 2.2. GFP Expressed from TRV2-GFP-PlHB31 in Infiltrated Organs

GFP fluorescence observations were preliminarily conducted in inoculated leaves to explore the optimal Agrobacterium solution concentration before infecting the whole rhizomes (Figure S2). Plants treated with “OD_600_ value = 1.5” exhibited much more intense, distinct, and stronger fluorescence signals than plants infected with “OD_600_ value = 1.2”, while, in wild-type plants, no signals were detected under the GFP field, which suggested that more cells expressed green fluorescence proteins in the infected organs. The abundance of GFP protein, which represented the concentration of TRV virus, implied that this was the appropriate Agrobacterium concentration for infecting herbaceous peony.

**Figure 2:**
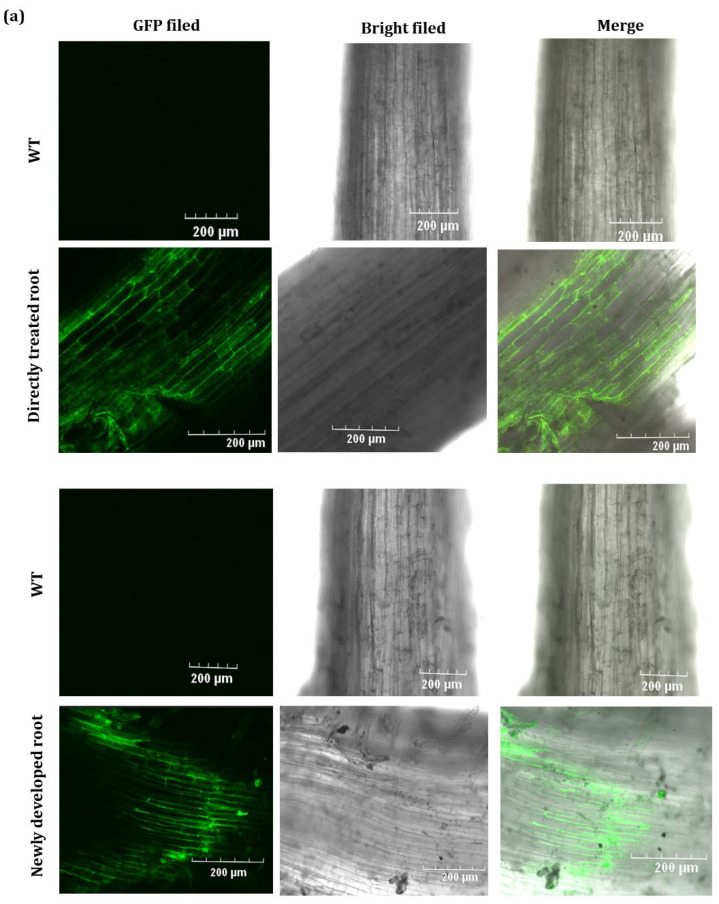

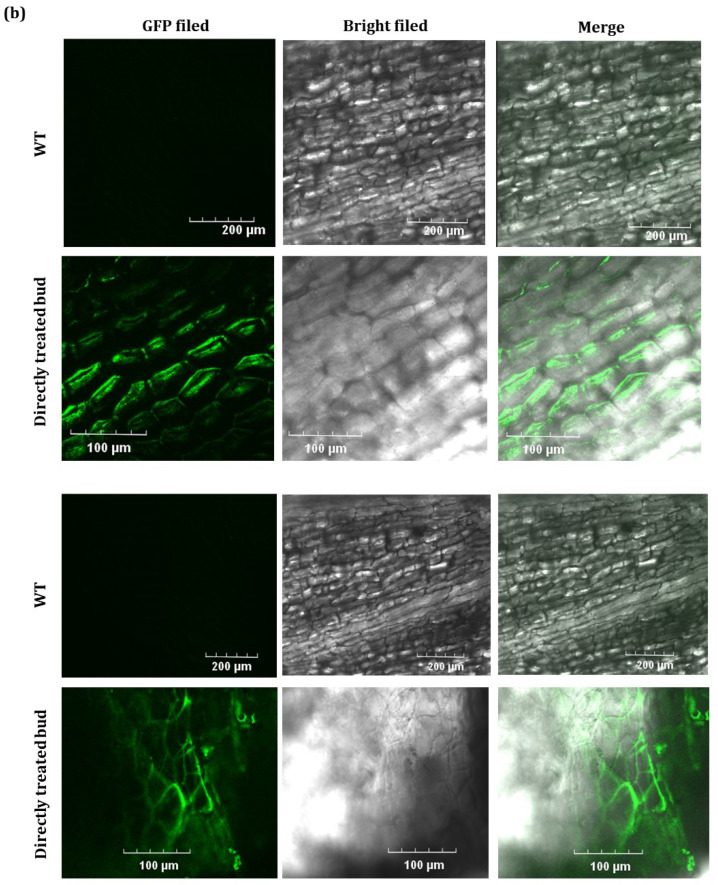
GFP expressed from infiltrated roots and buds in the TRV2-GFP-*PlHB31*-treated plants. The green fluorescence was observed under a laser scanning confocal microscope in directly treated and newly developed roots and buds at 3 d and 10 d post infection, respectively. (**a**) GFP expression in directly infiltrated roots and newly developed roots. (**b**) GFP expression in directly infiltrated buds and developed buds. Scale bars equal to 200 µm.

Green fluorescence was distinctly detected in infiltrated roots and buds to demonstrate the TRV’s ability to infect peony plants. The virus dynamics were successfully traced by fluorescence encoded by the EGFP tagged on the TRV2 vector. TRV2-GFP-*PlHB31* produced green fluorescence in the directly infiltrated roots and buds at 3 d post infection and moved to newly developed roots and buds at 10 d post infiltration, indicating that the TRV virus could spread in infiltrated and newly generated roots and developed buds. GFP fluorescence was not observed in wild-type roots and buds (Figure 2).

### 2.3. Screening and Silencing of PlHB31 in P. lactiflora

**Figure 3:**
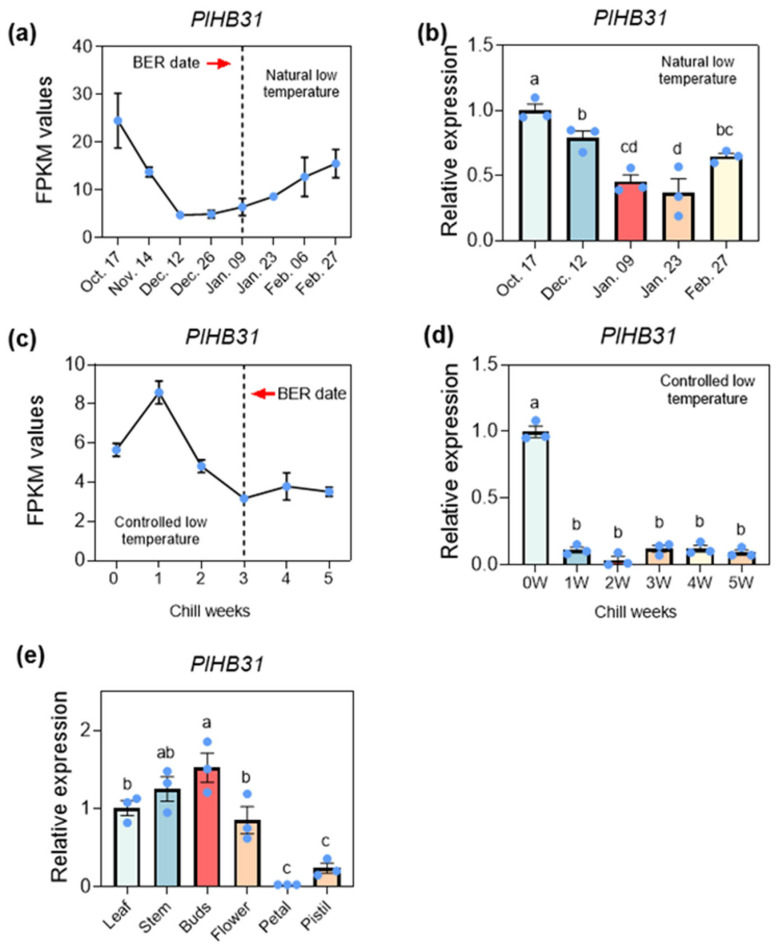
Expression of PlHB31 transcript and relative expression levels of *PlHB31* in *P. lactiflora* during BER under natural and controlled low temperatures. (**a**) Expression of *PlHB31* transcript under natural low temperature acquired from transcriptome data. (**b**) The expression levels of *PlHB31* acquired from qRT-PCR under natural low temperatures. (**c**) Expression of *PlHB31* transcript under controlled low temperature. (**d**) The expression levels of *PlHB31* acquired from qRT-PCR under controlled low temperatures. Dashed lines and arrows indicate BER dates. (**e**) The expression levels of *PlHB31* in different tissues of *P. lactiflora*. Error bars represent SEMs and different letters indicate significant differences (*p* < 0.05) determined by Student’s *t*-test.

According to expression of *PlHB31* transcript and qRT-PCR data, the expression levels of *PlHB31* in low-CR ‘Hang Baishao’ buds under a naturally low temperature decreased from 14 November to 26 December (endodormancy stage), and then increased in January and February (ecodormancy stage) (Figure 3a,b). The expression of *PlHB31* buds sharply decreased from week 0 to week 2 post controlled low temperature (endodormancy stage), and then slightly increased after week 3 and remained steady until week 5 (Figure 3c,d). Further, tissue-specific expression analysis showed that the expression of *PlHB31* was much higher in the buds and stems than in the leaves, petals, stamens, and sepals (Figure 3e). Thus, *PlHB31* is probably a negative regulator of BER in *P. lactiflora*, and it was chosen as template gene in this VIGS system construction.

**Figure 4:**
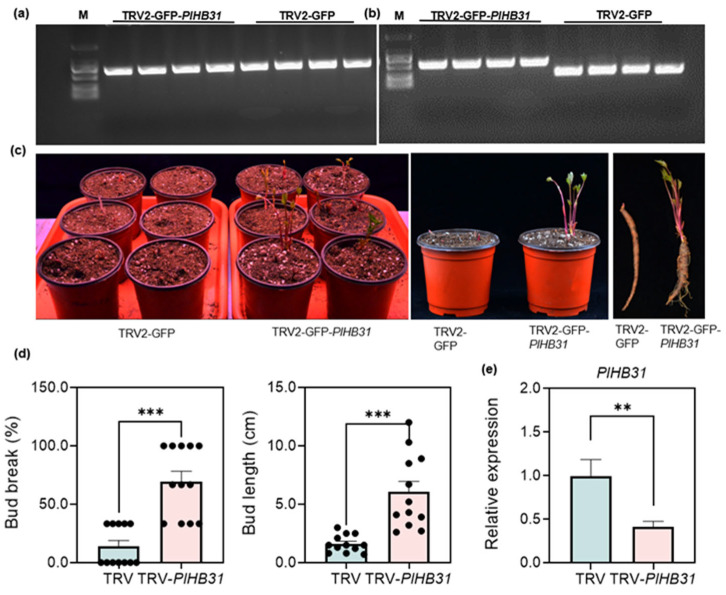
Detection of VIGS efficiency and phenotype differences between silenced and control plants. (**a, b**) RT-PCR was performed with locus-specific primers to the viral transcripts TRV1 and TRV2. M: 2000 DNA marker. (**a**) Band size of TRV1: 647 bp. (**b**) Band size of TRV2-GFP-*PlHB31* (555 bp) shows larger results due to the *PlHB31* insertion (274 bp), and band size of TRV2-GFP: 281 bp. (**c**) The phenotype of empty vector-transformed (TRV2-GFP) and TRV2-GFP-*PlHB31* silencing lines. (**d**) Plant morphological indices including bud-break rate and bud length of TRV2-GFP and TRV2-GFP-*PlHB31* silencing lines at 15 d post-infection. (**e**) The silencing efficiency of *PlHB31* was examined using q-RT PCR at 10 d post-infiltration in buds. Data in (**d**,**e**) are presented as mean ± SEM. Error bars represent the SEMs. Significant differences from the TRV2-GFP and TRV2-GFP-*PlHB31* silencing lines were determined by Student’s *t*-test (** *p* < 0.01, *** *p* < 0.001).

The silencing of *PlHB31* by a specific fragment was conducted in *P. lactiflora.* Specific primers for the TRV1 and TRV2 were designed to confirm the infection and the existence of the inserted fragment (Table 1). The TRV1 and TRV2 bands could be amplified from the *PlHB31*-silenced and TRV2-GFP plantlets. Furthermore, a bigger PCR product size was generated from TRV-GFP-*PlHB31*-treated plants as a result of *PlHB31′s* insertion into the TRV2-GFP vector (Figure 4a,b). Previous results of TRV2-GFP-*PlHB31* (Figure 2) and the previous detection of TRV1 and TRV2 transcripts in mock-treated and VIGS-treated plants (Figure 4a,b) provided sufficient evidence that silencing was due to the TRV-based vector treatment. *PlHB31*-silenced plants had a significantly early bud sprouting, increased sprouting rate, and increased bud length at two weeks after infiltration compared to the TRV2-GFP-transformed plants (Figure 4c,d). The bud-break rate of the mock-treated group was just 14%, whereas in the *PlHB31*-silenced group, it reached almost 70%, an almost five-fold increase in budbreak rate compared to that in the TRV-empty vector group. Additionally, bud length in the silenced group was significantly longer than that in the mock-treated group. Bud length could reach 12.0 cm in *PlHB31*-silenced plants, while the maximum length in mock-treated plants was only 3.0 cm. These results indicate that the TRV2-GFP-*PlHB31* successfully induced endogenous *PlHB31* silencing and promoted BER and bud growth in herbaceous peony. The underlying correlation between plantlet phenotype and *PlHB31* silencing was revealed at the gene expression level (Figure 4e). There was a significant downregulation of *PlHB31*, and silenced plants showed a 2.5-fold reduction in *PlHB31* mRNA transcript levels compared to mock-treated plants, indicating that *PlHB31* was successfully silenced by VIGS and that this silencing system was sufficiently efficient.

## 3. Discussion

### 3.1. VIGS Is an Ideal Technique for Identifying Gene Function in Herbaceous Peony but Needs to Be Improved Further

For most nonmodel plants, heterogenic verification is a frequently adopted method for gene function research that is carried out over a long period of time. Arabidopsis and tobacco are usually adopted to validate the function of genes associated with bud dormancy, stem growth, flower type, color formation, and stress tolerance in peonies [30,31,32,33]. The gene functional annotation and expression patterns observed from omics data were also regarded as auxiliary references when speculating on gene function in peonies. Wide acceptance of these conclusions is more difficult than that of results obtained by homologous verification using peony plants themselves, which seriously inhibits further discovery of the mystery of peonies [34].

Homologous verification techniques for identifying gene functions in plants and animals have been developed for several decades; however, most techniques have not yet been applied to peonies. Gene overexpression, RNA interference, and CRISPR/Cas9 are extremely difficult to realize using in vitro organs of peonies due to the lack of an efficient genetic transformation system [35]. The callus of peonies inevitably encounters browning and vitrification problems despite meticulous operation and sterilization. Moreover, explant cultures of peony are subject to various types of contamination. The efficiency of multiple-shoot and root inductions is also limited, which leads to difficulties in generating integrated plantlets in vitro. Additionally, the poor transplantation and acclimation of tissue culture seedlings causes a low survival rate after their removal from culture bottles [12,13,14,15]. Consequently, gene silencing via VIGS has gradually become the only efficient strategy to realize transient homologous verification in peonies.

VIGS does not alter nucleotide sequences or generate offspring with heritable traits; it only induces posttranscriptional gene silencing within a limited period. Therefore, VIGS does not need a stable and prolonged genetic transformation system and has gradually become the only alternative approach in peony research to identify the function of genes associated with flower color formation, stem strength, dwarfing, thermotolerance, and disease resistance [28,29,30,31,32,33]. The procedures of this technique only include pretreatment, plasmid construction, bacterial solution preparation, infection, and post-cultivation, without complicated tissue culture and with no requirement for stable genetic transformation. Therefore, VIGS is a convenient, time-saving, and easily controlled method for research on gene function in herbaceous peonies. However, two problems in the VIGS system are in urgent need of improvement before its use in peonies, i.e., realizing intact-plant infiltration and minimizing its operation.

### 3.2. Selection One-Year-Old Roots of Herbaceous Peony for VIGS: Realizing the Intact-Plant Infiltration and Achieving the Minimization of the Operation Space, Material, Reagent and Manpower

**Figure 5:**
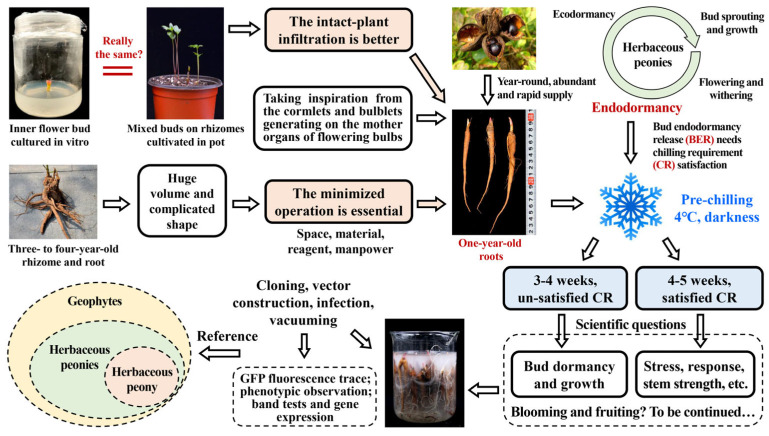
Idea conception, highlights, and reference significance of this homologous silencing system in herbaceous peony.

Theoretically, the totipotency of plant cells ensures that any organ of a plant can be grown into an intact plant under suitable conditions. Therefore, the explants, after Agrobacterium infection and subsequent in vitro culture, could grow and develop into whole transgenic plantlets based on their cell totipotency, efficient tissue culture, and genetic transformation systems [36]. Silencing plants’ in vitro organs is not the best strategy for gene function identification, especially in dormancy research. For tree peony (chamaephytes), when inner flower buds with their outer scales removed are cultivated in MS medium, their dormancy and germination are not necessarily equivalent to the dormancy and sprouting of mixed buds generated in the rhizomes of intact plants [28]. For herbaceous peonies (geophytes), the dormancy and sprouting of underground buds without direct exposure to sunlight generated in the underground roots of intact plants are different from the dormancy and germination of flower buds removed from inside the mixed bud and cultured in medium with fixed illumination (Figure 5). Intact-plant infiltration could achieve a more realistic and dependable silencing effect compared with infection of part of the plant organs. Therefore, intact plants in their natural state are ideal and prioritized materials for VIGS. In this study, one-year-old roots of herbaceous peony were selected as the materials for VIGS, inspired by the cormlet and bulblet used as materials for VIGS in bulbous flowers, to successfully achieve intact-plant infiltration, which is an innovation of this system.

Three- to four-year-old roots have been utilized for VIGS in herbaceous peonies in a few studies [23,24,25,26,27]. In most cases, the length of three- to four-year-old roots is approximately 20–40 cm, and the diameter is approximately 5–8 cm; furthermore, such roots generally have several branches, numerous fibrils, and 3–10 mixed buds of large sizes and complex and irregular shapes. Therefore, cleaning and infection are time-, water- and reagent-consuming; moreover, they require adequately large operation space in the laboratory. Comparatively, one-year-old roots have delicate sizes, spindly shapes, nearly no branches, average lengths of approximately 8.5–12.5 cm, and diameters of approximately 1.0–1.5 cm (Figure 5). Therefore, a total of 3–10 one-year-roots could be planted in a one-gallon small flowerpot, and 40 one-year-old roots could simultaneously be submerged in a 500-mL beaker. Such a system could lead to marked savings in operation space, manpower, reagents, and various experimental materials, as well as achieving a good infection efficiency and acceptable contamination rates.

In addition, one-year-old roots have the potential to be used as a material for transient verification in the following two research fields: first, the function of the genes involved in regulating bud dormancy, sprouting, and other growth processes could be validated by infecting roots. With no aboveground parts, the underground root is the only existing organ of herbaceous peony that exists in winter. Hence, gene functions could be distinctly identified by the speed and ratio of bud sprouting and growth. Second, the function of genes related to stem length, stress response, and resistance could also be demonstrated via root infection and silencing [23,24,25,26]. Stress treatments such as heat, chilling, drought, stem-pulling, salt, and alkali could be conducted once a root grows to a whole plant, and gene function could be validated based on morphological performance (Figure 5).

### 3.3. Ensuring a Year-Round Abundant Supply of Robust Materials for Infection and Silencing

The year-round availability of infection materials is the third advantage of choosing one-year-old roots. The seeds of herbaceous peony, stored at −20 °C, could maintain their vitality, and could then be rejuvenated after being potted and cultured in a growth chamber [37]. Thereafter, one-year-old roots could be obtained within half a year. Low-temperature storage and a short growth cycle could both contribute to a steady and abundant supply of one-year-old roots without seasonal limitations and ensure that the VIGS experiment could be conducted at any time (Figure 5).

One-year-old roots are very young herbaceous peony plants that are sufficiently healthy and have less disease and rot, and fewer insect pests, than three- to four-year-old roots [38]. Therefore, one-year-old roots could provide robust and vigorous materials for infection and silencing.

### 3.4. Determination of the Crucial Prechilling Treatment Duration Depends on Specific Scientific Questions

Subjecting roots to a chilling treatment before conducting a VIGS experiment is an essential precondition because roots must achieve a certain chilling accumulation, i.e., satisfy their CR, to complete BER [39]. According to our previous research, the Utah model (UT model) was proven to be the best model to evaluate the CR of herbaceous peony. The optimal prechilling temperature is 4 °C, which could provide one chill unit (CU), whereas temperatures in other ranges would provide lower or even negative CU values for herbaceous peony. The potted roots of ‘Hang Baishao’ were treated at 4 °C for at least 4 weeks to obtain a sufficient chilling-to-release bud endodormancy and trim sprouting [37,40].

In the current VIGS system, the duration of prechilling at 4 °C is flexible and can be adjusted for different research purposes. If the research topic is bud dormancy and the target gene is relevant to bud dormancy regulation, the prechilling duration could be adjusted to 3–4 weeks; if the research topic is the stress response, resistance, stem strength, and photosynthetic response, among others, the prechilling duration in a 4 °C refrigerator could be adjusted to 4–5 weeks (Figure 5). To be more precise, for bud dormancy regulation research, 3–4 weeks is an applicable chilling duration because the CRs of most herbaceous peony cultivars are unsatisfactory after 3–4 weeks at 4 °C, and these cultivars could not completely break bud endodormancy [40,41]. Bud sprouting and growth performances will show a significant difference between silenced and control plantlets. Notably, roots, after four or more weeks of chilling, acquire sufficient chilling accumulation and then meet the CR. Therefore, both silenced and control plantlets could sprout easily, without distinguishable phenotypic differences, even after gene silencing. In fact, 4–5 weeks of prechilling is suitable for other studies, such as those on environmental responses, stress resistance, and stem strength, among other topics. Most herbaceous peony cultivars with CR fulfillment could perform uniform bud sprouting and growth after 4–5 weeks of chilling duration, which is the prerequisite for subsequent stress treatment after gene silencing [42] (Figure 5).

In addition to prechilling treatment duration, the infection time, the concentration of Agrobacterium, pre-pricking on roots, and post infection-cultivation are also worthy of attention. In this study, the penetration effect of Agrobacterium was insufficient with a vacuuming time of less than 10 min; conversely, too long an infection time would probably lead to rot and the disintegration of fleshy shoots and roots [23,24,25]. Agrobacterium solution with OD_600_ = 1.5 was the appropriate concentration for herbaceous peony. A low value of OD_600_ would decrease infection efficiency, while an excessively high value of OD_600_ would lead to plantlet wilt or even death, since one-year-old rhizomes are delicate and tender [26,27]. Post-infection cultivation also plays a key role, since most herbaceous peony species are long-day plants, so they need sufficient illumination to make sure normal burgeon and growth.

### 3.5. Functional Identification of the Template Gene PlHB31 via the Established VIGS System

In most studies, *PHYTOENE DESATURASE* (*PDS*) was identified as a key enzyme in the biosynthesis of protective carotene and is commonly used as a silencing indicator during VIGS system establishment. The silencing of *PDS* by infecting leaves, stems, and petals causes an easily recognizable photobleaching phenotype [43,44]. However, we attempted to silence *PlPDS* many times, but it was difficult for new leaves and stems to exhibit photobleaching. The color of leaves and stems of herbaceous peony is dark purple at the early developmental stage, which lasts for a long period, and changes to green at the late developmental stage, which results from a reduction in anthocyanins and the deposition of chlorophylls [45]. Therefore, bleaching in tender leaves and stems induced by *PlPDS* silencing after bud sprouting was imperceptible in the intact-plant infiltration using dormant roots in this study. The reason for this finding is possibly the overabundance of anthocyanin in tender leaves and stems; additionally, *PDS* mainly participates in carotenoid biosynthesis instead of anthocyanin biosynthesis [46]. According to our repeated attempts, *PDS* is not suitable for checking the success of VIGS system construction using dormant one-year-old roots.

*PlHB31*, a member of the homeodomain with leucine zipper (HD-Zip) proteins, was proven to activate dormancy-associated MADS-box 1 (*DAM1*) to regulate bud dormancy release in pear [47]. The HD-Zip gene family is important for plant development and growth and has been widely investigated in Arabidopsis, cotton, and tomato, among other plant species [48,49]. As revealed by the transcriptome data, the expression of *PlHB31* showed a decreasing trend from paradormancy to endodormancy during the BER period, indicating that this gene probably plays a negative regulatory role in responding to BER in herbaceous peony. The expression of *PlHB31* under natural and controlled low temperatures and in different tissues in *P. lactiflora* further indicated the inhibitor role of *PlHB31* during BER (Figure 3). After infection, the qRT-PCR results showed that the transcript abundance of *PlHB31* was significantly downregulated in the silenced plantlets. (Figure 4e), which was consistent with the expectations and phenotypic performance.

TRV2-GFP was able to infect many species, producing GFP and moving systematically throughout plant cells [50,51,52]. As the indicator gene, GFP is easier to track in herbaceous peony. In the GFP images, the TRV vector could be clearly monitored by a confocal microscope, whereas no fluorescence signal was detected in the control plants (Figure 2), suggesting that the recombinant TRV2-GFP vector can efficiently replicate and spread systemically in herbaceous peony. In conclusion, morphological differences in bud sprouting and stem growth, differences in *PlHB31* expression between the silenced and control groups, and the detection of GFP in different tissues could prove the successful establishment of this VIGS system, with intact-plant infiltration and minimized operation.

### 3.6. The Established System Is Suitable for Research on the Functions of Genes Regulating Various Physiological Activities, except Blooming and Fruiting, in Herbaceous Peony

The established transient verification system presented in this study is valuable and efficient for unraveling most molecular mechanisms, such as dormancy, growth, development, photosynthesis, and respiration, as well as various environmental responses and stress resistance in herbaceous peony. Nonetheless, research on blooming and fruiting using this VIGS system is limited due to the inherent characteristics of one-year-old roots [53]. Although the one-year-old roots of peonies have many irreplaceable advantages in gene function research, they are still in the juvenile phase and are unable to bloom and fruit after bud sprouting. Therefore, the function of genes related to flower color and form regulation, fragrance formation, and oilseed production could not be explored using this VIGS system.

Notably, the silencing effect induced by VIGS was only sustained for approximately one month because VIGS is a transient verification technique [54,55]. The one-year-old roots of herbaceous peony could sprout within two weeks and finish all vegetative growth within one month as long as the prechilling supply was sufficient. In addition, a series of key processes, such as phenotypic observation, data collection, sampling, and multiple treatments, could be completed in one month, before the decrease or disappearance of the silencing effect. However, most cultivars of herbaceous peony need more than one month to bloom and a much longer cultivation period to obtain fruits, which means that transient verification through root infection is probably inapplicable to blooming- and fruiting-related research [40].

### 3.7. The Established VIGS System Is Suitable for Gene Function Research in All Herbaceous Peonies and Provides a Reference for Gene Silencing in Other Geophytes

The VIGS technique is especially valuable in species where genetic transformation is challenging or time-consuming, especially in geophytes that grow from bulbs, corms, tubers, rhizomes, and tuberous root. Previous research focusing on the construction of the VIGS system yielded optimal results and shed light on the transient transformation in geophytes [56,57]. Inspired by the cormlet and bulblet used as materials for VIGS to investigate gene regulation in bud growth transitions and corms dormancy in bulbous flowers, we established a homologous silencing system with intact-plant infiltration and minimized operation based on a prior study on VIGS system construction in herbaceous and tree peonies [44,58,59]. This established methodology is also applicable to more than 30 species of herbaceous peonies and can provide a valuable reference for all other geophytes. All these species could produce one-year-old underground organs generated from seeds or mother organs, which would provide sufficient and equivalent infection materials for this VIGS system, and these organs would theoretically be applicable to intact-plant infiltration and gene silencing. In conclusion, the VIGS system established in this study is of high value for scientific research on the herbaceous peony *P. lactiflora*, approximately 30 species of herbaceous peonies, and all other geophytes (Figure 5).

## 4. Materials and Methods

### 4.1. Plant Material and Selection Standard

**Figure 6:**
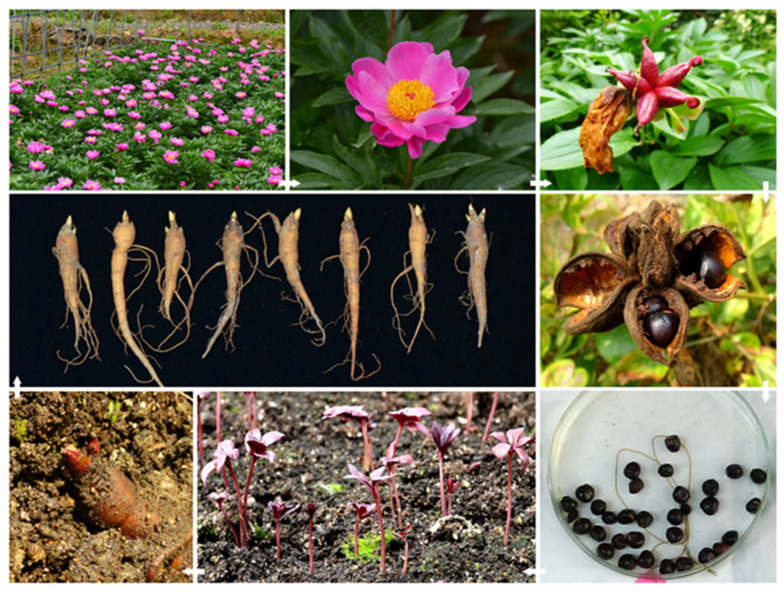
Acquirement of one-year-old roots of *P. lactiflora* ‘Hang Baishao’.

*P. lactiflora* ‘Hang Baishao’, a typical low-latitude herbaceous peony, was selected as the material to construct the VIGS system. This cultivar was introduced from Pan’an County (E 120°17′–120°47′, N 28°49′–29°19′), Zhejiang Province, China. As a native southern cultivar in a low-latitude region with more than one thousand years of cultivation history, ‘Hang Baishao’ has beautiful pink flowers (Figure 6), indigenous adaptability, special low-CR trait/short bud endodormancy duration, and strong resistance to multiple stresses, as revealed in our previous multiyear studies [39].

Seeds of ‘Hang Baishao’ were harvested from mature fruits in late summer and stored at 4 °C in a refrigerator, and could be sown at any time. One-year-old roots were generated three to four months after sowing and then adopted as crucial infection materials. The intact structure of a one-year-old root includes the top rhizome (sometimes called as crown in peonies), rhizome buds, spindly main root, and adventitious roots (Figure 6). Because all these organs would be immersed by Agrobacterium solution in the infection process, they were uniformly called “one-year-old roots” in this study in order to describe them concisely. In general, the parameters of the selected one-year-old roots were as follows: length, 8.5–12.5 cm; diameter, 1.0–1.5 cm; 1–3 robust dormant buds; and plentiful white fibrous roots (Figure 6).

### 4.2. Prechilling Treatment

One-year-old roots were temporarily potted with peat soil and perlite, mixed at a ratio of 1:2, and then continuously treated at 4 °C in darkness and 65% humidity for 3–4 or 4–5 weeks to reach a certain chilling accumulation [40,41]. The prechilling treatment duration depends on the specific research objectives: 3–4 weeks for bud dormancy, budbreak, and growth research, and 4–5 weeks for research on stress resistance, environmental response, stem strength, and photosynthesis, among other processes. The reasons for this will be elaborated in the Discussion. The prechilled roots were rinsed with distilled water and dried in air before infection with Agrobacterium solution [27].

### 4.3. Target Gene Identification and Isolation

Total RNA from dormant buds of ‘Hang Baishao’ cultivated in a nursery garden was extracted with the TIANGEN RNA Prep Pure Plat kit (Tiangen, Beijing, China) and then conserved at −80 °C. The first strand of cDNA was synthesized using the Prime Script RT Reagent Kit with gDNA Eraser (Takara, Shiga, Japan). According to the analyses of transcriptome data, including the differentially expressed transcript analysis and weighted gene co-expression network analysis, and expression patterns revealed by qRT-PCR under natural and controlled low temperatures, *HOMEOBOX PROTEIN 31* (*PlHB31*) was chosen as the template gene with a 932 bp full-length sequence. Specific primers were designed with a 274 bp target fragment (Table 1). A polymerase chain reaction (PCR) was conducted to acquire the amplified coding sequence of *PlHB31*, and the procedure was as follows: 35 cycles of 98 °C for 1 min, 98 °C for 10 s, 55 °C for 15 s, and 72 °C for 15 s, and a final cycle of 72 °C for 10 min.

### 4.4. Vector Construction

**Figure 7:**
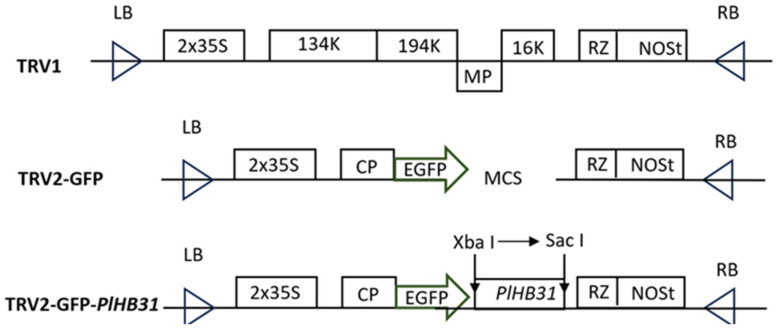
Schematic representation of TRV1, TRV2-GFP, and TRV2-GFP-*PlHB31.* LB: left border, RB: right border, MP: movement protein, 16K: 16 Kd protein, RZ: self-cleaving ribozyme, NOSt: NOS terminator, CP: coat protein, MCS: multiple cloning site.

The TRV2-GFP empty vector with EGFP-tagged coat protein (CP) fragment (Figure 7) exhibited similar efficiency in infection and gene silencing to TRV2 vector [51]; then, it was linearized using restriction enzymes (*Xba I* and *Sac I*) at 37 °C for 30 min in this study. The linearized vector was then linked with the isolated *PlHB31* fragment using a Vazyme One Step Cloning Kit (Takara, Shiga, Japan), and the product was identified by agarose gel electrophoresis and purified using a Mini BEST Agarose Gel Extraction kit (Takara, Shiga, Japan). The purified recombinant plasmid was transformed into 100 μL of *Escherichia. coli DH5α* competent cells (Bioteke, Beijing, China) and then incubated in Luria–Bertani (LB) liquid medium for 1 h (37 °C, 200 rpm); thereafter, competent cells were selected on LBK plates containing 50 mg/L kanamycin after 12–16 h of incubation (37 °C, 200 rpm, in darkness). Monocolonies were inoculated into fresh LB medium, and plasmids were extracted for further DNA sequence alignment to verify the correct insertion of the fragment.

### 4.5. Plasmid Transformation and Preparation for Infection

TRV1, TRV2-GFP, and TRV2-GFP-*PlHB31* plasmids were transformed into *Agrobacterium* strain GV3101 (Weidibio, Shanghai, China) via the freeze-thaw method [55]. Transformant bacteria were cultured and selected in LB plates containing 50 mg/L kanamycin and 50 mg/L rifampin at 28°C for 16 h. PCR was conducted to examine the presence of the inserted *PlHB31* fragment using the following procedure: 30 cycles of 94 °C for 5 min, 94 °C for 30 s, 57 °C for 30 s, and 72 °C for 30 s, and a final cycle of 72 °C for 10 min.

**Figure 8:**
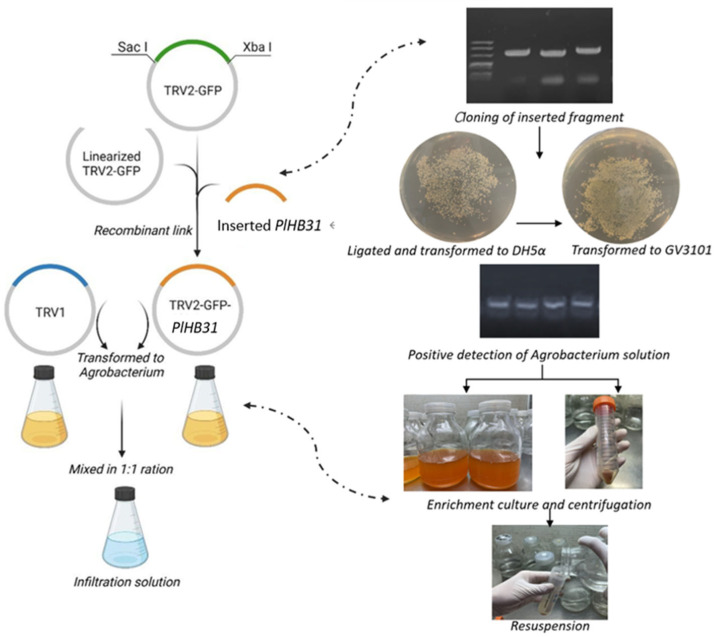
Schematic diagram of the recombination of TRV2-GFP-*PlHB31* and the preparation of infiltration solution.

The bacteria were then cultured in LB liquid medium supplemented with 50 mg/mL kanamycin, 50 mg/mL rifampin, 10 mM 2-morpholinoethanesulfonic acid (MES), and 20 µM acetosyringone (AS) at 28 °C and 200 rpm for 16 h. The Agrobacterium cells were then centrifuged at 4000 rpm for 10 min at 25 °C after the OD_600_ value of bacterial solution finally reached 1.5. The retained Agrobacterium strains were then resuspended in 500 mL of infection buffer containing 10 mM MgCl_2_, 10 mM MES, and 20 µM AS and cultured at 28 °C in the dark for 3 h. Equal volumes of TRV1 and TRV2-GFP or TRV1 and TRV2-GFP-*PlHB31* were mixed at a ratio of 1:1 to obtain the final infection solution (Figure 8).

Before vacuuming infection of whole rhizomes, the fully expanded leaves of one-year-old *P. lactiflora* were preliminarily inoculated to confirm the final OD_600_ value for more efficient infection. Different concentrations of Agrobacterium solution were injected into leaves using a 1 mL needleless syringe. Then, inoculated plantlets were cultured for 3 days in darkness at 20 °C. The occurrence of fluorescence could be roughly identified by an ultraviolet lamp and distinctly observed using a laser-scanning confocal microscope (TCS SP8, Leica, Wetzlar, Germany).

### 4.6. Vacuum Infiltration of the Intact-Plant with One-Year-Old Roots

**Figure 9:**
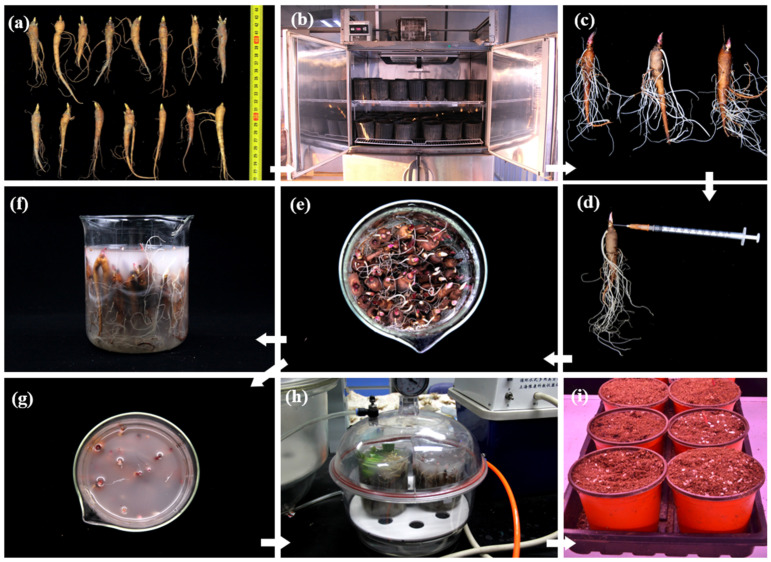
Schematic illustration of a homologous transient system involving VIGS for one-year roots of herbaceous peony. (**a**) Standardized roots with a length of 8.5–12.5 cm and 1–3 buds. (**b**) Prechilling treatment at 4 °C for 2–3 weeks. (**c**) Rinsing and draining. (**d**) Pricking on the base of buds and top of fleshy root. (**e**–**g**) Roots were fully submerged in infection solution in a 500 mL beaker. (**h**) Vacuum pumping for 10 min and deflating for 20 min. (**i**) Post-infection culturation in a growth chamber.

After rinsing and drying, prechilled one-year-old roots were poked with one pinhole on the base of the buds and two pinholes in the middle of the fleshy roots. The whole roots were then submerged completely in infiltration solution, subjected to negative pressure vacuum-pumping (−0.9 kg/cm^2^) (SHB-IIIA, Shanghai Yetuo Technology, Shanghai, China) for 10 min, and slowly deflated for 20 min [23,24,25,26,27]. A total of 40 roots could be immersed in each 500 mL beaker simultaneously based on the small size and regular spindle shape of one-year-old roots. The number of immersed roots could be increased by increasing the volumes or numbers of beakers and vacuum pumps. Thereafter, post-infected roots were rinsed, dried, potted, and cultured at 20 °C in darkness for 3 days. The potted roots were then transferred to a growth chamber with a temperature of 25 °C, 65% humidity, 16 h light/8 h dark cycle, and 4000-lux illumination (Figure 9).

### 4.7. GFP Imaging Detection

The infected plantlets were cultured in darkness for 3 days at 20 °C and transferred to a growth chamber at 24 °C with 60% humidity under a 16 h light/8 h dark cycle. GFP imaging in infiltrated leaves, roots, and bud cells of *P. lactiflora* was conducted using a laser scanning confocal microscope (TCS SP8, Leica). Green fluorescence in inoculated leaves, directly treated roots, and buds at 3 days post-infection, and green fluorescence in newly developed roots and buds at 10 days post-infiltration, were observed. GFP signals in infected organs were excited by the 488 nm laser line and were optimally detected by a 510 nm laser line.

### 4.8. Phenotypic Observation

Phenotype observation is a visual and reliable method for identifying gene function. Ecodormancy was nonexistent because the environment in the growth chamber was suitable for bud sprouting and root growth. Therefore, BER could be observed when the top of the bud emerged from the soil and tender leaves emerged from bud scales after cultivation in a growth chamber for 7–10 days. The plant morphology, such as budbreak rate and bud length, was observed.

For bud dormancy research, phenotypic data would support the validation of gene function in dormancy regulation, whereas for other research topics, such as stress resistance, environmental response, stem strength, and photosynthesis, phenotypic observation would be conducted after the plantlets were uniformly treated (approximately 10–15 days after potting) with different gradients of heat, drought, illumination, and pulling, among others.

### 4.9. Determination of Gene Expression

Total RNA was extracted from tender stems and leaves of silenced and control plantlets to validate the success of the established VIGS system. The first-strand cDNA was then synthesized from 1 µg of total RNA using the Prime Script™ RT Reagent Kit with gDNA Eraser (Takara, Shiga, Japan). Specific primer pairs were designed to detect the presence of TRV2-GFP-*PlHB31* and TRV1 using Reverse Transcriptase (RT)-PCR (Table 1). The PCR products were then analyzed via agarose gel electrophoresis detection. The band length of the amplified products of the silenced plantlets should be longer than that of the control plantlets when using primers for TRV2, whereas the same length should be obtained using primers for TRV1. Quantitative real-time (q-RT) PCR was then carried out using TB Green™ Premix Ex Taq™ II (Takara, Shiga, Japan) in a system with a total volume of 20 µL to calculate the efficiency of the transformation. Alpha-tubulin (ATUBA) was used as a reference gene; the relative expression levels in the silenced and control plantlets were analyzed using a CFX96 Real-Time PCR Detection System (Bio-Rad, Hercules, CA, USA). The expression patterns of *PlHB31* under natural and controlled low temperatures were also analyzed by q-RT PCR. Data were calculated via the 2^−ΔΔCt^ method.

## Figures and Tables

**Table 1:** Specific primers designed for construction of the recombinant TRV vector and PCR amplification.

Primer Names	Nucleotide Sequences (5′-3′)	Purposes	Sizes
*PlHB31*	F: AAGGTTACCGAATTCTCTAGA CTGCAACTGCCACAGGAACT R: GGCCTCGAGACGCGTGAGCTC TGTCAGTATCTGATCCGCCG	Cloning inserted fragment and q-RT PCR analysis	247 bp
TRV1	F: AAGGTTACCGAATTCTCTAGA TTACAGGTTATTTGGGCTAG R: GGCCTCGAGACGCGTGAGCTC CCGGGTTCAATTCCTTATC	RT-PCR analysis	647 bp
TRV2	F: AAGGTTACCGAATTCTCTAGA TGGGAGATGATACGCTGTT R: GGCCTCGAGACGCGTGAGCTC CCTAAAACTTCAGACACG	RT-PCR analysis (containing inserted fragment)	>281 bp

## Data Availability

The datasets generated during and/or analyzed during the current study are available from the corresponding author on reasonable request.

## Supplementary Materials

The following supporting information can be downloaded at: https://www.mdpi.com/article/10.3390/ijms25084412/s1.

## References

[1] Liu W., Yuan J.S., Stewart C.N. (2013). Advanced genetic tools for plant biotechnology. Nat. Rev. Genet..

[2] Zhang Y., Liang Z., Zong Y., Wang Y., Liu J., Chen K., Qiu J.-L., Gao C. (2016). Efficient and transgene-free genome editing in wheat through transient expression of CRISPR/Cas9 DNA or RNA. Nat. Commun..

[3] Butt H.I., Yang Z.E., Gong Q., Chen E.Y., Wang X.Q., Zhao G., Ge X.Y., Zhang X.Y., Li F.G. (2017). *GaMYB85*, an R2R3 MYB gene, in transgenic Arabidopsis plays an important role in drought tolerance. BMC Plant Biol..

[4] Zhang K.L., Yao L.J., Zhang Y., Baskin J.M., Baskin C.C., Xiong Z.M., Tao J. (2019). A review of the seed biology of Paeonia species (Paeoniaceae), with particular reference to dormancy and germination. Planta.

[5] Kamenetski R., Barzilay A., Cohen M. Herbaceous peony for cut flower production: Flowering physiologation techniques and cultivation techniques. Proceedings of the International Conference on Quality Management in Supply Chains of Ornamentals, King Mongkuts Univ Technol Thonburi.

[6] Falavigna V.D.S., Guitton B., Costes E., Andres F. (2019). I Want to (Bud) Break Free: The Potential Role of *DAM* and *SVP-Like* Genes in Regulating Dormancy Cycle in Temperate Fruit Trees. Front. Plant Sci..

[7] Miotto Y.E., Tessele C., Czermainski A.B.C., Porto D.D., Falavigna V.D., Sartor T., Cattani A.M., Delatorre C.A., de Alencar S.A., da Silva O.B. (2019). Spring Is Coming: Genetic Analyses of the Bud Break Date Locus Reveal Candidate Genes from the Cold Perception Pathway to Dormancy Release in Apple (*Malus × domestica Borkh*.). Front. Plant Sci..

[8] Kamenetsky-Goldstein R., Yu X. (2022). Cut peony industry: The first 30 years of research and new horizons. Hortic. Res..

[9] Barzilay A., Zemah H., Kamenetsky R., Ran I. (2002). Annual life cycle and floral development of ‘*Sarah Bernhardt*’ peony in Israel. Hortscience.

[10] Zhang J., Zhang D., Wei J., Shi X., Ding H., Qiu S., Guo J., Li D., Zhu K., Horvath D.P. (2019). Annual growth cycle observation, hybridization and forcing culture for improving the ornamental application of *Paeonia lactiflora* Pall. in the low-latitude regions. PLoS ONE.

[11] Ji L.J., Wang Q., da Silva J.A.T., Yu X.N. (2012). The genetic diversity of *Paeonia* L.. Sci. Hortic..

[12] Duan S.Y., Xin R.J., Guan S.X., Li X.T., Fei R.W., Cheng W., Pan Q., Sun X.M. (2022). Optimization of callus induction and proliferation of *Paeonia lactiflora* Pall. and Agrobacterium-mediated genetic transformation. Front. Plant Sci..

[13] Cai X., Wei H., Liu C., Ren X.X., Luc H., Jeong Y.R.Y. (2020). Synergistic Effect of NaCl Pretreatment and PVP on Browning Suppression and Callus Induction from Petal Explants of *Paeonia Lactiflora* Pall. ‘Festival Maxima’. Plants.

[14] Shen M.M., Tang Z.J., da Silva J.A.T., Yu X.N. (2015). Induction and proliferation of axillary shoots from in vitro culture of *Paeonia lactiflora* Pall. mature zygotic embryos. N. Z. J. Crop Hortic. Sci..

[15] Shen M.M., Wang Q., Yu X.N., da Silva J.A.T. (2012). Micropropagation of herbaceous peony (*Paeonia lactiflora* Pall.). Sci. Hortic..

[16] Liu Y.L., Schiff M., Dinesh-Kumar S.P. (2002). Virus-induced gene silencing in tomato. Plant J..

[17] Liu Q.L., Xu K.D., Yi L., Hou Y.L., Li D.X., Hu H.Y., Zhou F., Song P.W., Yu Y.G., Wei Q.C. (2021). A rapid, simple, and highly efficient method for VIGS and in vitro-inoculation of plant virus by INABS applied to crops that develop axillary buds and can survive from cuttings. BMC Plant Biol..

[18] Burch-Smith T.M., Anderson J.C., Martin G.B., Dinesh-Kumar S.P. (2004). Applications and advantages of virus-induced gene silencing for gene function studies in plants. Plant J..

[19] Purkayastha A., Mathur S., Verma V., Sharma S., Dasgupta I. (2010). Virus-induced gene silencing in rice using a vector de-rived from a DNA virus. Planta.

[20] Liu N., Xie K., Jia Q., Zhao J., Chen T., Li H., Wei X., Diao X., Hong Y., Liu Y. (2016). Foxtail Mosaic Virus-Induced Gene Silencing in Monocot Plants. Plant Physiol..

[21] Li Y., Kong F., Liu Z., Peng L., Shu Q. (2022). PhUGT78A22, a novel glycosyltransferase in Paeonia ‘He Xie’, can catalyze the transfer of glucose to glucosylated anthocyanins during petal blotch formation. BMC Plant Biol..

[22] Sun Y., Shang W., Yuan J., Wang Z., He S., Song Y., Shi L., Shen Y., Ma J., Xu Y. (2022). Functional Analysis of *PsARRO−1* in Root Development of *Paeonia suffruticosa*. Horticulturae.

[23] Tang Y.H., Lu L.L., Huang X.Q., Zhao D.Q., Tao J. (2023). The herbaceous peony transcription factor WRKY41a promotes secondary cell wall thickening to enhance stem strength. Plant Physiol..

[24] Wang X., Li J., Guo J., Qiao Q., Guo X., Ma Y. (2020). The WRKY transcription factor PlWRKY65 enhances the resistance of *Paeonia lactiflora* (herbaceous peony) to *Alternaria tenuissima*. Hortic. Res..

[25] Zhao D.Q., Luan Y.T., Shi W.B., Tang Y.H., Huang X.Q., Tao J. (2022). Melatonin enhances stem strength by increasing lignin content and secondary cell wall thickness in herbaceous peony. J. Exp. Bot..

[26] Qian Y., Cheng Z., Meng J., Tao J., Zhao D. (2023). PlMAPK1 facilitates growth and photosynthesis of herbaceous peony (*Paeonia lactiflora* Pall.) under high-temperature stress. Sci. Hortic..

[27] Bian T., Ma Y., Guo J., Wu Y., Shi D., Guo X. (2020). Herbaceous peony (*Paeonia lactiflora* Pall.) PlDELLA gene negatively regulates dormancy release and plant growth. Plant Sci..

[28] Zhang Y., Gao L., Wang Y., Niu D., Yuan Y., Liu C., Zhan X., Gai S. (2023). Dual functions of *PsmiR172b-PsTOE3* module in dormancy release and flowering in tree peony (*Paeonia suffruticosa*). Hortic. Res..

[29] Gao L., Niu D., Chi T., Yuan Y., Liu C., Gai S., Zhang Y. (2023). PsRGL1 negatively regulates chilling- and gibberellin-induced dormancy release by PsF-box1-mediated targeting for proteolytic degradation in tree peony. Hortic. Res..

[30] Chen X., Duan X., Wang S., Wu W., Zhang X. (2019). Virus-induced gene silencing (VIGS) for functional analysis of MYB80 gene involved in *Solanum lycopersicum* cold tolerance. Protoplasma.

[31] Sun J., Wu Y., Shi M., Zhao D., Tao J. (2020). Isolation of *PlANS* and *PlDFR* genes from herbaceous peony (*Paeonia lactiflora* Pall.) and its functional characterization in Arabidopsis and tobacco. Plant Cell Tissue Organ Cult..

[32] Zhang Y., Xu S., Cheng Y., Wang J., Wang X., Liu R., Han J. (2020). Functional identification of *PsMYB57* involved in an-thocyanin regulation of tree peony. BMC Genet..

[33] Meng J., Guo J., Li T., Chen Z., Li M., Zhao D., Tao J. (2022). Analysis and Functional Verification of *PlPM19L* Gene Associated with Drought-Resistance in *Paeonia lactiflora* Pall. Int. J. Mol. Sci..

[34] Zulfiqar S., Farooq M.A., Zhao T.T., Wang P.P., Tabusam J., Wang Y.H., Xuan S.X., Zhao J.J., Chen X.P., Shen S.X. (2023). Virus-Induced Gene Silencing (VIGS): A Powerful Tool for Crop Improvement and Its Advancement towards Epigenetics. Int. J. Mol. Sci..

[35] Abudayyeh O.O., Gootenberg J.S., Essletzbichler P., Han S., Joung J., Belanto J.J., Verdine V., Cox D.B.T., Kellner M.J., Regev A. (2017). RNA targeting with CRISPR-Cas13. Nature.

[36] Silva J.A.T.d., Shen M., Yu X. (2012). Tissue Culture and Micropropagation of Tree Peony (*Paeonia suffruticosa* Andr.). J. Crop Sci. Biotechnol..

[37] Wang X.B., Li D.Q., Zhang D., Shi X.H., Wu Y., Qi Z.Y., Ding H.Q., Zhu K.Y., Xia Y.P., Zhang J.P. (2020). Improving crucial details and selecting the optimal model for evaluating the chilling requirement of *Paeonia lactiflora* Pall. at low lati-tudes during four winters. Sci. Hortic..

[38] Ni W., Zhao H., Xu W., Xu F., Huang S., Wang R., Wu D. (2023). First Report of Root Rot Caused by Pleiocarpon algeriense on Peony (*Paeonia suffruticosa*) in China. Plant Dis..

[39] Zhang J.P., Li D.Q., Shi X.H., Zhang D., Qiu S., Wei J.F., Zhang J., Zhou J.H., Zhu K.Y., Xia Y.P. (2017). Mining and expression analysis of candidate genes involved in regulating the chilling requirement fulfillment of *Paeonia lactiflora* ‘Hang Baishao’. BMC Plant Biol..

[40] Wang X.B., Zhang R.L., Huang Q.Y., Shi X.H., Li D.Q., Shao L.M., Xu T., Horvath D.P., Xia Y.P., Zhang J.P. (2022). Comparative Study on Physiological Responses and Gene Expression of Bud Endodormancy Release Between Two Her-baceous Peony Cultivars (*Paeonia lactiflora* Pall.) with Contrasting Chilling Requirements. Front. Plant Sci..

[41] Zhang R.L., Wang X.B., Shi X.H., Shao L.M., Xu T., Xia Y.P., Li D.Q., Zhang J.P. (2021). Chilling Requirement Validation and Physiological and Molecular Responses of the Bud Endodormancy Release in *Paeonia lactiflora* ‘Meiju’. Int. J. Mol. Sci..

[42] Park J.H., Rhie Y.H., Lee S.Y., Kim K.S. (2015). Pre-chilling Promotes Flowering in *Paeonia lactiflora* ‘Taebaek’ without Flower Bud Abortion. Hortic. Environ. Biotechnol..

[43] Kumagai M.H., Donson J., Dellacioppa G., Harvey D., Hanley K., Grill L.K. (1995). Cytoplasmic Inhibition of Carotenoid Biosynthesis with Virus-Derived RNA. Proc. Natl. Acad. Sci. USA.

[44] Xie L., Zhang Q., Sun D., Yang W., Hu J., Niu L., Zhang Y. (2019). Virus-induced gene silencing in the perennial woody *Paeonia ostii*. PeerJ.

[45] Tang Y., Fang Z., Liu M., Zhao D., Tao J. (2020). Color characteristics, pigment accumulation and biosynthetic analyses of leaf color variation in herbaceous peony *Paeonia lactiflora* Pall.). 3 Biotech.

[46] Zhang Y., Xu S., Ma H., Duan X., Gao S., Zhou X., Cheng Y. (2021). The R2R3-MYB gene *PsMYB58* positively regulates an-thocyanin biosynthesis in tree peony flowers. Plant Physiol. Biochem..

[47] Yang Q.S., Niu Q.F., Li J.Z., Zheng X.Y., Ma Y.J., Bai S.L., Teng Y.W. (2018). *PpHB22*, a member of HD-Zip proteins, activates *PpDAM1* to regulate bud dormancy transition in ‘Suli’ pear *Pyres pyrifolia* White Pear Group). Plant Physiol. Biochem..

[48] Gao Y., Gao S., Xiong C., Yu G., Chang J., Ye Z., Yang C. (2015). Comprehensive analysis and expression profile of the homeodomain leucine zipper IV transcription factor family in tomato. Plant Physiol. Biochem..

[49] Chen Q., Sun M., Zhao G., Yang F., Long X., Li J., Wang J., Yu Y. (2017). Origin of the mafic microgranular enclaves (MMEs) and their host granitoids from the Tagong pluton in Songpan-Ganze terrane: An igneous response to the closure of the Paleo-Tethys Ocean. Lithos.

[50] Zhong X.H., Yuan X., Wu Z., Khan M.A., Chen J., Li X.X., Gong B.H., Zhao Y., Wu J., Wu C. (2014). Virus-induced gene silencing for comparative functional studies in *Gladiolus hybridus*. Plant Cell Rep..

[51] Tian J., Pei H., Zhang S., Chen J., Chen W., Yang R., Meng Y., You J., Gao J., Ma N. (2014). TRVGFP: A modified Tobacco rattle virus vector for efficient and visualizable analysis of gene function. J. Exp. Bot..

[52] Tian J., Cheng L., Han Z.-y., Yao Y.-c. (2015). Tobacco rattle virus mediated gene silencing in strawberry plants. Plant Cell Tissue Organ Cult..

[53] Halevy A.H., Barzilay A., Kamenetsky R. Flowering advancement in herbaceous peony. Proceedings of the 9th International Symposium on Flower Bulbs.

[54] Ryu C.M., Anand A., Kang L., Mysore K.S. (2004). Agrodrench: A novel and effective Agroinoculation method for virus-induced gene silencing in roots and diverse Solanaceous species. Plant J..

[55] Cheng G.H., Shu X.C., Wang Z., Wang N., Zhang F.J. (2023). Establishing a Virus-Induced Gene Silencing System in *Lycoris chinensis*. Plants.

[56] Singh A., Kumar P., Jiang C.Z., Reid M.S. (2013). TRV Based Virus Induced Gene Silencing in *Gladiolus grandiflorus* L.), A Monocotyledonous Ornamental Plant. Vegetos.

[57] Pan W.Q., Li J.R., Du Y.P., Zhao Y.J., Xin Y., Wang S.K., Liu C., Lin Z.M., Fang S.Z., Yang Y.D. (2023). Epigenetic silencing of callose synthase by VIL1 promotes bud-growth transition in lily bulbs. Nature Plants.

[58] Wu J., Jin Y.J., Liu C., Vonapartis E., Liang J.H., Wu W.J., Gazzarrini S., He J.N., Yi M.F. (2019). GhNAC83 inhibits corm dormancy release by regulating ABA signaling and cytokinin biosynthesis in *Gladiolus hybridus*. J. Exp. Bot..

[59] Guan S.X., Kang X.N., Ge J.Y., Fei R.W., Duan S.Y., Sun X.M. (2022). An efficient Agrobacterium-mediated transient transformation system and its application in gene function elucidation in *Paeonia lactiflora* Pall. Front. Plant Sci..

