# Supplementary figures and images for: Establishment of a Homologous Silencing System with Intact-Plant Infiltration and Minimized Operation for Studying Gene Function in Herbaceous Peonies
Source: Int J Mol Sci. 2024 Apr 17;25(8):4412. doi: 10.3390/ijms25084412 (PMC11050706; doi:10.3390/ijms25084412)

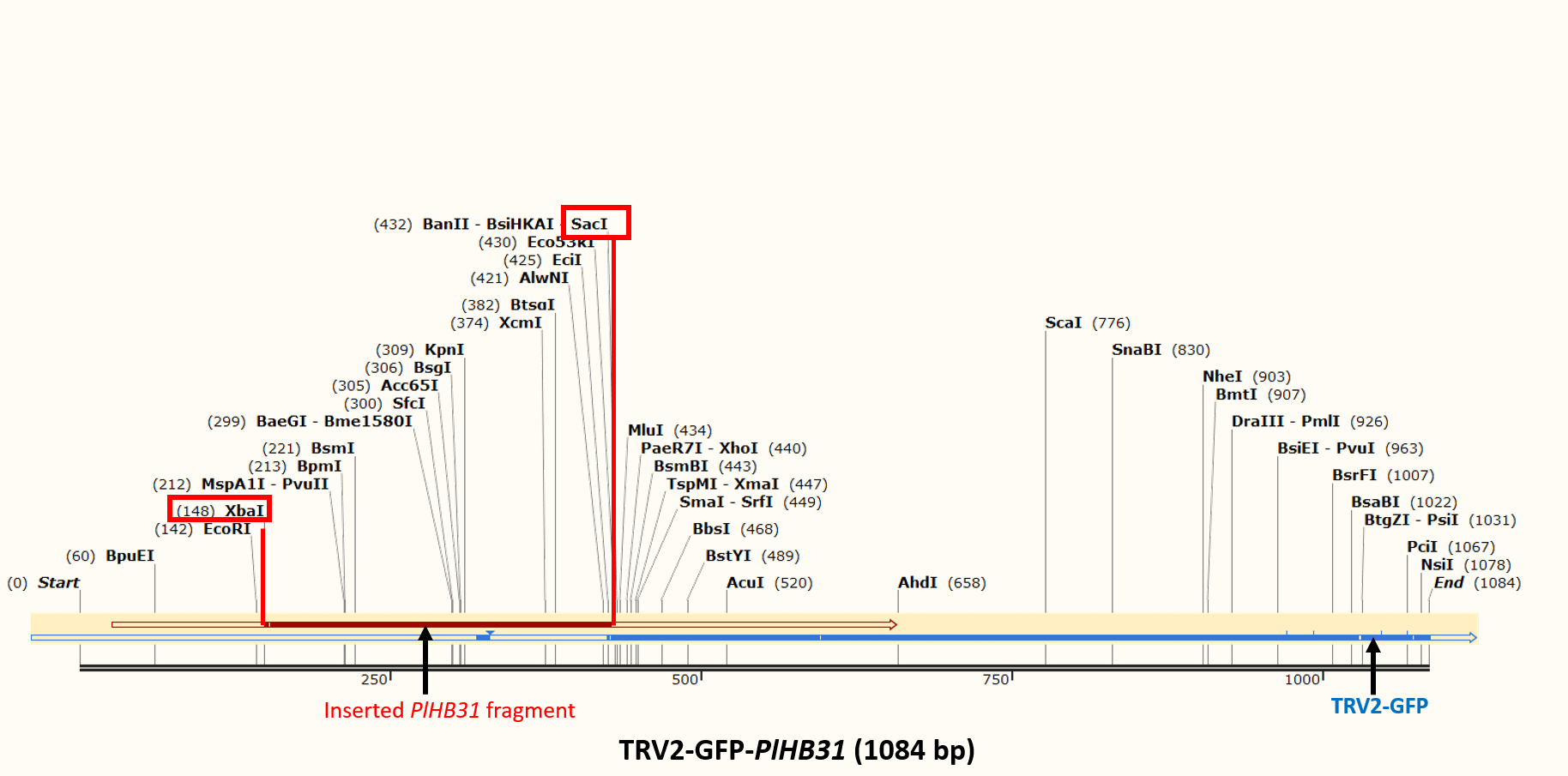

Supplement: Supplementary file 1 [file ijms-25-04412-s001.zip › Figure S1.png]

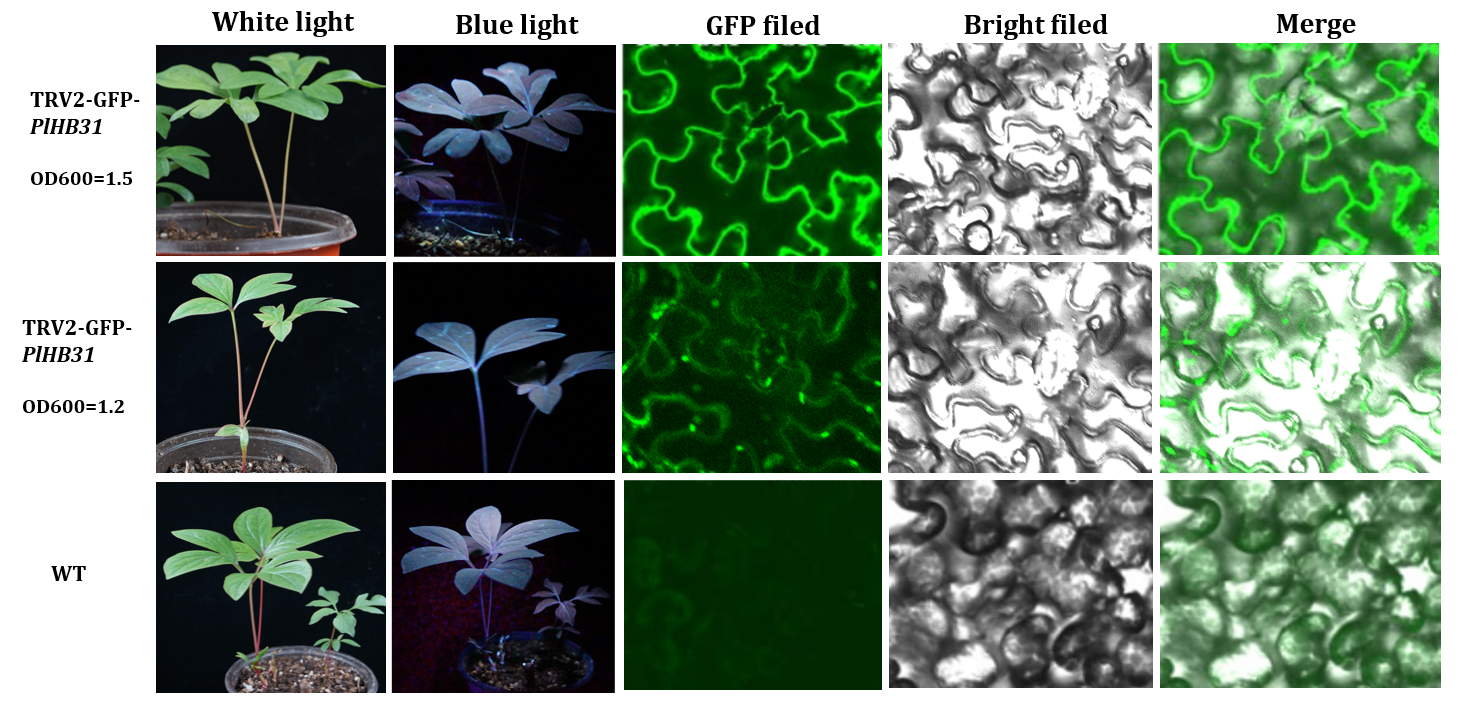

Supplement: Supplementary file 1 [file ijms-25-04412-s001.zip › Figure S2.png]
